# *Leishmania* spp. in indigenous populations: A mini-review

**DOI:** 10.3389/fpubh.2022.1033803

**Published:** 2022-12-22

**Authors:** Louise Bach Kmetiuk, Thais Cristina Tirado, Leandro Meneguelli Biondo, Alexander Welker Biondo, Fabiano Borges Figueiredo

**Affiliations:** ^1^Graduate Program in Biosciences and Biotechnology, Instituto Carlos Chagas, Fundação Oswaldo Cruz, Curitiba, Paraná, Brazil; ^2^Reference Laboratory for Leishmaniasis, Instituto Carlos Chagas, Fundação Oswaldo Cruz, Curitiba, Paraná, Brazil; ^3^National Institute of the Atlantic Forest (INMA), Brazilian Ministry of Science, Technology, and Innovation, Santa Teresa, Espírito Santo, Brazil; ^4^Department of Veterinary Medicine, Federal University of Paraná (UFPR), Curitiba, Paraná, Brazil

**Keywords:** vulnerability, zoonoses, leishmaniasis, one health, vector-borne diseases

## Abstract

Leishmaniasis, considered a neglected vector-borne disease complex of global concern, has a significant impact on indigenous communities due to daily human and animal exposure in periurban, rural, and naturally preserved areas. This mini-review aims to assess and discuss studies of leishmaniasis in these communities of the New World and Old World, particularly those in the Americas and Asia. Such indigenous communities have been mostly built in poor traditional households with no mosquito-net protection, mostly located in environmentally protected areas, favoring vectors and reservoirs. The presence of leishmaniasis cases surrounding such indigenous areas indicated a high risk of infection, which may have been historically underestimated due to a lack of surveillance, even at present. The absence of studies of indigenous populations in recognized endemic areas may reflect insufficient health services. In conclusion, the persistence of this neglectful scenario may impact tragic outcomes and potential outbreaks in indigenous peoples and surroundings populations worldwide.

## Introduction

Leishmaniasis has been described as a neglected vector-borne disease complex caused by an obligatory protozoan of the genus *Leishmania*, with human and animal infections spread by parasite-infected sandflies (1, 2). Depending on the parasite species, three main clinical forms of leishmaniasis have been naturally observed: cutaneous leishmaniasis (CL), mucocutaneous leishmaniasis (MCL), and visceral leishmaniasis (VL) (3). Reservoirs may perpetuate the *Leishmania* life cycle by acting as a source of transmission to vectors and may include canids, rodents, marsupials, hyraxes, and occasionally humans (4). Approximately 98 species of *Phlebotomu*s and *Lutzomyia* sandflies have been suggested or confirmed as vectors of 53 *Leishmania* species worldwide (4, 5).

The genus *Leishmania* probably evolved in the Mesozoic era before splitting the supercontinent Pangaea (6). The particular geographical origin of the different species of *Leishmania* remains controversial, with at least three currently plausible hypotheses (6). The genus *Lutzomyia* probably emerged after the isolation of *Phlebotomus* in the Americas, with the subgenera *Nyssomyia, Psychodopygus*, and *Lutzomyia* s. str. acting as the main vectors of *Leishmania* spp. (5). *Lutzomyia* sandfly distribution remained primarily limited to preserved and anthropized forest areas of tropical areas and both VL and CL in the Americas, mostly associated with sylvatic habitats and peridomestic transmission (5).

Climatic, environmental, and socioeconomic factors have been associated with the occurrence of leishmaniasis (7). Although it has been challenging to conclusively establish the evolution of *Leishmania* spp. as zoonotic parasites in the New World and Old World, the association has generally been related to the origin of humans in eastern Africa and subsequent movements in Asia, Africa, Europe, and the Americas (5, 6). Overall, leishmaniases have been reported as endemic in tropical, subtropical, and Mediterranean areas, comprising 83 countries or regions, with 350 million people at risk and 0.9 and 1.7 million people infected yearly (8). Despite being reported in 98 countries in Europe, Africa, Asia, and the Americas, over 90% of new cases are found in just 13 countries: Afghanistan, Algeria, Bangladesh, Bolivia, Brazil, Colombia, Ethiopia, India, Iran, Peru, South Sudan, Sudan, and Syria (6). The disease has been considered absent to date in Australia, New Zealand, and the southern Pacific (5).

**Figure 1:**
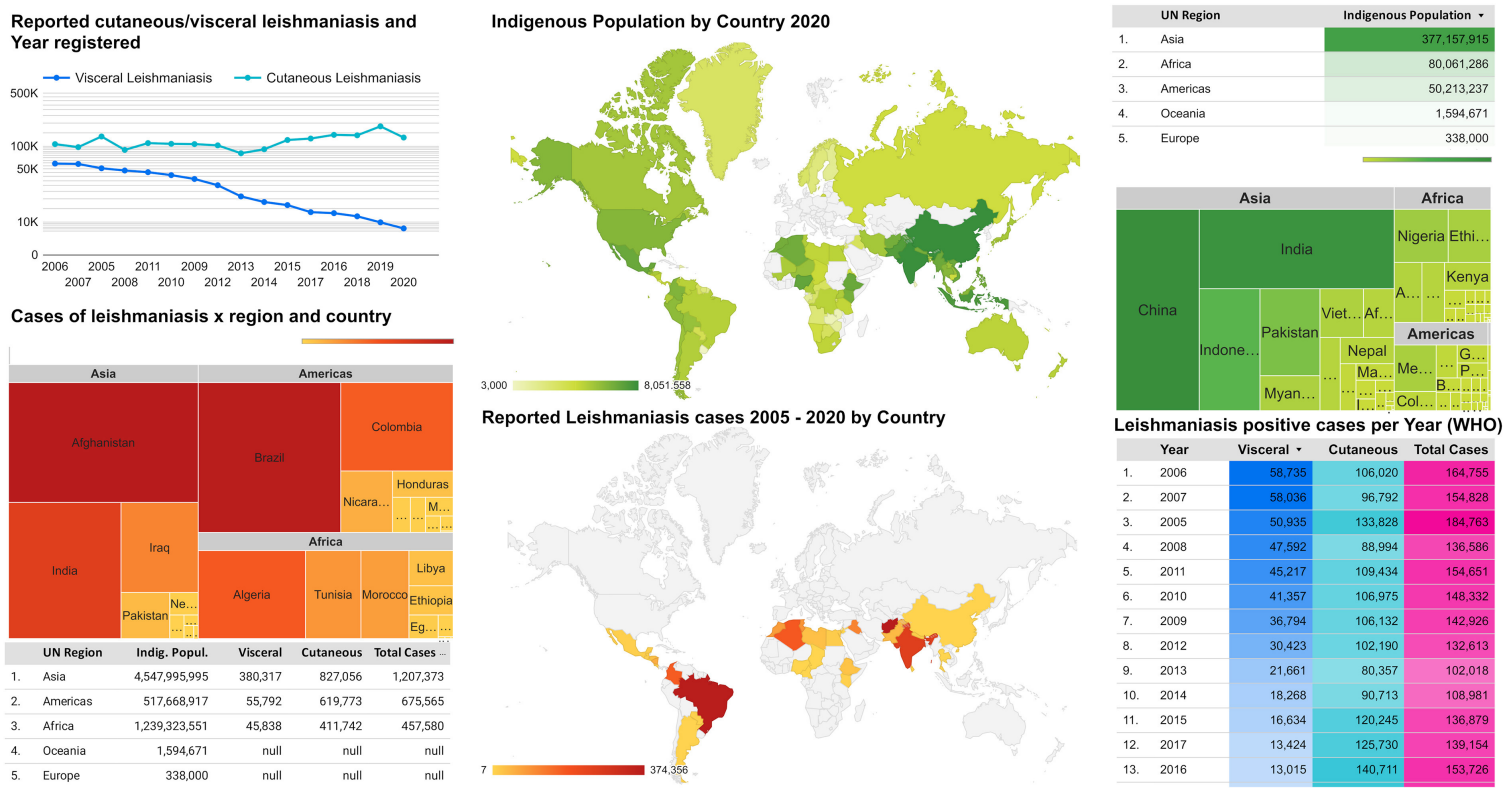
Global distribution of leishmaniasis endemic areas and the average of indigenous populations.

Although the first autochthonous cases of cutaneous and mucocutaneous leishmaniasis in the Americas were reported in 1909, visceral leishmaniasis only erupted as an American public health concern in 1934 (9). The first reports of ovoid bodies in a spleen smear were noted in 1900 by the Scottish pathologist William Boog Leishman (source name of genus *Leishmania*) while serving the British Army in India (6). Leishmaniasis went on to become the only neglected tropical disease in the world whose outlook worsened over the decades. Brazil represented over 97% of the VL cases in the Americas by 2020 (8), while Colombia, Brazil, Costa Rica, and Peru, along with African countries, represent 70–75% of new CL cases globally (10). The distribution of recent cases of human leishmaniasis strongly overlaps with indigenous populations, particularly in tropical and subtropical areas (Figure 1). The presence of leishmaniasis cases surrounding indigenous areas may indicate a high risk of infection, which may have been historically underestimated due to a lack of surveillance, even at present. Most new leishmaniasis cases worldwide have been reported in countries with indigenous communities located within endemic areas (Figure 1). Unfortunately, indigenous areas worldwide mostly overlap leishmaniasis distribution, aggravated by the concomitant lowest domestic general government investment in health per country and population proportion pushed below US$ 3.20 found in these tropical and subtropical areas (Supplementary Figure 1). Moreover, mean temperatures and average annual rain precipitation have provided favorable environmental conditions in such areas (Supplementary Figure 2).

Therefore, vulnerable human populations such as indigenous communities may be exposed within both periurban and remote areas near preserved areas (11). Moreover, since sandflies and *Leishmania* spp. have become adapted to peridomestic characteristics and domestic reservoirs (7), indigenous people may be increasingly exposed due to the overlapping of endemic areas with their communities (Supplementary Figures 1, 2). Accordingly, the present study aims to summarize CL, MCL, and VL occurrence in indigenous populations, gathering and analyzing the frequency and epidemiological data.

## Methods

A comprehensive review was conducted using a geographic approach as a background for historical, epidemiological, and diagnostic information. It summarized indexed studies on CL, MC, and VL in the indigenous population and their animals. Data for the review were primarily obtained using PubMed and Google Scholar as the main database and the MeSH terms “leishmania indigenous population” OR “leishmania indigenous people” OR “leishmaniasis indigenous population” OR “leishmaniasis indigenous people.”

Following the removal of duplicates and the screening of titles and abstracts, an assessment of the full texts was thoroughly performed for inclusion or exclusion criteria. To be included, studies must assess an indigenous population and/or their dogs, as well as vectors for the detection of anti-*Leishmania* spp. antibodies or parasitological or molecular diagnosis; geographical and language restrictions were not applied.

Reviews, systematic reviews, meta-analyses, and other articles that failed to meet the inclusion criteria were removed from the present study, particularly those using the term “indigenous” to describe autochthonous cases in nonindigenous populations, and antileishmanial activity of medicinal plants.

The analysis of economic and social indicators presented herein within the figures and supplementary data were produced using extracted data from The World Bank (12), also available on Google Cloud public datasets, accessible with Google BigQuery and the World Development Indicators (WDI) collection (Supplementary material 1). From the WDI collection, 84 indicators with data from Brazil were filtered but used only those indicators no older than five and with at least 10 years of the complete database. A geographic map of countries and leishmaniasis occurrence was then compared to distribution maps from the remaining WDI indicators. Those related to general poverty and health may be visually related to disease maps. Selected indicators “Proportion of population pushed below the $3.20 ($ 2011 PPP) poverty line by out-of-pocket health care expenditure (%)” and “Domestic general government health expenditure (% of GDP)” were then named SH.UHC.NOP2.ZS and SH.XPD.GHED.GD.ZS, respectively. The first has shown the fraction of the country's population experiencing out-of-pocket health impoverishing expenditures with data from 1996 to 2017, and the second had data for health expenditures from 2000 to 2019 updated on Global Health Expenditure Database (GHED) efforts. These two variables were selected to represent both the economical capacity of countries to invest in health and their population poverty condition pushed by private expenditures to overcome the lack of public health availability and visually related to leishmaniasis. Finally, the WorldBank visual representation of treated data for Mean Sea Level Temperature and Total Annual Precipitation was used for referencing the climate relation with leishmaniasis and other neglected tropical diseases as per the World Health Organization (WHO).

## Results

### Study characteristics

The initial bibliographical survey found a total of 96 scientific articles, 15 of which were included in the present review following the screening on the inclusion criteria. The selected articles were conducted between 1989 and 2019 in five different countries, with 9/15 (60.0%) from Brazil, 3/15 (20.0%) from Colombia, and 1/15 (6.6%) each from Guyana, Iran, and Taiwan. As such, 13/15 (86.6%) were conducted in the Americas and 2/15 (13.3%) in Asia (Table 1, Supplementary Table 1, Supplementary Figure 3).

**Table 1:** *Leishmania* spp. and related vector in indigenous individuals and dogs worldwide.

	***Leishmania* species involved**	**Diagnostic method/** **sasmpling**	**Frequency**	**Recognized or potential vector**	**Comment(s) (including related disease[s])**	**Year/ geographical distribution- country/ references**
Human beings	*L. (L.) chagasi*	Isoenzyme analysis of cultured parasites/bone marrow aspirate, splenic aspirate RIFI/ serum	82 cases of VL	*Lu. Longipalpis*	Fever, anemia, diarrhea, and hepatosplenomegaly as associated clinical signs.	1989–1993/ America- Brazil (28)
	*Leishmania* (*Viannia*) spp.	PCR (kDNA)/skin lesion scarification	27/30 (90.0%)	-	Cutaneous form with one (22/30; 73.0%) or more lesions (8/30; 27.0%), majority in males (73%).	2016–2018/ America- Brazil (30)
	*L. infantum*	PCR cathepsin L-like gene/serum	3/372 (0.8%)	*Lu. longipalpis; Lutzomyia cruzi*	-	NA/ America- Brazil (20)
	*Leishmania* sp.	Giemsa staining/ montenegro's intradermal test	-	-	Congenital heart disease associated with severe malnutrition	2019/ America- Brazil (22)
	*L. chagasi*	Leishmania skin test (LST). ELISA; IFAT/serum	73/385,19.0% (LST); 23/454, 5.1% (ELISA); 21/454, 4.6% (IFAT)	*Lutzomyia cayannensis*; *Lu. longipalpis*	No associated clinical signs.	1995–1996/ America- Colombia (34)
	*L. infantum*	IFAT/serum	0/580 (0.0%)	*-*	-	2006/ America- Colombia (28)
	*Leishmania* spp.	Microscopic examination/ lesion smear	274/539 (50.8%)	*Lutzomya (Nyssomyia) trapidoi*; *Lu. panamensis*	-	2010–2014/ America- Colombia (34)
	*Leishmania tropica*	Microscopic examination or histological analysis/ lesion smear	1085/1250 (86.8%)	*Phlebotomus ansari; Phlebotomus papatasii; Phlebotomus caucasicus, and Phlebotomus sergenti*	Lesions are less severe and acute among indigenous.	1990–1992/ Asia- Iran (65)
	*L. tropica*	RT-PCR/ fresh frozen skin tissue	4 cases of CL	*-*	-	2005/ Asia-Taiwan (66)
	*L. tropica*	Microscopic examination or histological analysis/ lesion smear	1085/1250 (86.8%)	*Phlebotomus ansari; Phlebotomus papatasii; Phlebotomus caucasicus, and Phlebotomus sergenti*	Lesions are less severe and acute among indigenous.	1990–1992/ Asia- Iran (31)
	*L.infantum*	RIFI/ serum	390/3.773 (10.4%)	*Lu. longipalpis*	-	1989–1993/ America- Brazil (28)
	*Leishmania orientalis* (previously described as *Leishmania siamensis*)	Isolation on culture medium and identification by PCR/ bone marrow	1^*^	*Sergentomyia iyengari* (69)	Human immunodeficiency virus (HIV) infected patient	2010/ Asia- Thailand (59)
	*Leishmania orientalis*	Microscopic examination/ skin biopsy	1^*^	*Sergentomyia iyengari* (69)	Confirmed case of cutaneous leishmaniasis caused by *L. orientalis*	2014/ Asia- Thailand (56)
	*Leishmania orientalis* (previously described as *Leishmania siamensis*)	Isolation on culture medium and identification by PCR/ skin biopsy	1^*^	*Sergentomyia iyengari* (69)	Human immunodeficiency virus (HIV) infected patient with disseminated cutaneous leishmaniasis. Presence of mild splenomegaly by ultrasonography.	2015/ Asia- Thailand (70)
	*Leishmania* sp.	Microscopic examination of smears and PCR/ bone mar- row	1^*^	*Sergentomyia* spp. *and Phlebotomus* spp. (71–73)	Confirmed case of visceral leishmaniasis in non-HIV-infected patient	2006/ Asia- Thailand (60)
	*L. martiniquensis* (previously described as ‘*Leishmania siamensis*')	Microscopic examination of smears/ bone mar- row	1^*^	*Sergentomyia* spp. and *Culicoides* spp. (71–73)	Confirmed case of visceral leishmaniasis in a 5-year-old child, with hepatomegaly and recurrence after amphotericin B treatment.	2008/ Asia- Thailand (74)
	*Leishmania* sp.	Microscopic examination of smears and PCR/ bone mar- row	1^*^	*Sergentomyia* spp. and *Culicoides* spp. (71–73)	Human immunodeficiency virus (HIV) patient co-infected with tuberculosis and visceral leishmaniasis, presenting prolonged fever, anemia, thrombocytopenia, hepatomegaly, and nephritonephrotic syndrome.	2010/ Asia- Thailand (75)
	*Leishmania martiniquensis*	qPCR/ blood, buffy coat, urine, and saliva	10 individuals^*^	*Sergentomyia* spp. and *Culicoides* spp. (71–73)	A total of 8/10 (80.0%) patients were co-infected with HIV, and 4/10 (40.0%) were affected with both CL and VL.	2013/ Asia- Thailand (76)
	*Leishmania* sp.	Isolation on culture medium and identification by PCR/ bone marrow, ulcer, urine and oral fluid samples	2^*^	*Sergentomyia* spp. and *Culicoides* spp. (71–73)	Human immunodeficiency virus (HIV) patients with cutaneous and visceral leishmaniasis	2011/ Asia- Thailand (77)
	*L. martiniquensis*	Microscopic examination, culture, isolation and PCR/ bone marrow	1^*^	*Sergentomyia* spp. and *Culicoides* spp. (71–73)	Confirmed case of visceral leishmaniasis in non-HIV-infected patient with subacute fever, huge splenomegaly and pancytopenia	2012/ Asia- Thailand (78)
	*L. martiniquensis*	Microscopic examination, culture, isolation and PCR/ skin and bone marrow	2^*^	*Sergentomyia* spp. and *Culicoides* spp. (71–73)	Cutaneous and visceral leishmaniasis in HIV-infected patients presenting chronic generalized fibrotic skin lesions	2012/ Asia- Thailand (79)
	*L. martiniquensis* (previously described as ‘*Leishmania siamensis*')	Microscopic examination, culture, and PCR/ skin biopsy	1^*^	*Sergentomyia* spp. and *Culicoides* spp. (71–73)	Disseminated dermal leishmaniasis in a systemic steroid therapy patient.	2013/ Asia- Thailand (58)
	'*L. martiniquensis'; 'L. siamensis'*	PCR; microscopic observation/ saliva; ulcer or nodule	3/316 (0.95%)	*Sergentomyia* spp. and *Culicoides* spp. (71–73)	Presence of disseminated cutaneous lesions and nodular lesions in positive saliva test patients	2013/ Asia- Thailand (80)
(72)	*L. martiniquensis* (previously described as ‘*Leishmania siamensis*')	Microscopic examination, culture, and PCR/ nodules biopsy	1^*^	*Sergentomyia* spp. and *Culicoides* spp. (71–73)	Human immunodeficiency virus (HIV) patients with multiple cutaneous nodules on his brow and fingers	2014/ Asia- Thailand (81)
Dogs	*Leishmania* (*L*.) *chagasi*	ELISA and IFA/Serum restriction fragment length polymorphism (RFLP PCR)/ tissue samples of seropositive euthanized dogs	29/63 (46.0%)	*-*	Splenomegaly, wasting, onychogryphosis, opaque fur, and hepatomegaly in 18.2% of positive dogs. Most dogs were asymptomatic (27.3%) and oligosymptomatic (54.5%).	2007/ America- Brazil (27)
	*L. infantum*	IFA/ Serum	185/270 (68.5%).	*-*	-	2006/ America- Colombia (35)
	*L. infantum*	IFA/ Serum	1/20 (5.0%)	*-*	Dog seropositive for *L. infantum* was acquired from Waiwai communities in Northwest Brazil, the possibility of previous infection.	2019/ America- Guyana (37)

NA, not available;

* autochthons case of non-indigenous/ non-aboriginal individuals.

## Discussion

### Leishmaniasis in indigenous peoples of New World

The New World occurrence of *L. infantum* has generally been associated with the European colonization of the Americas in the late 1400s, with the disease distribution ranging from the southern United States to northern Argentina (5, 13). As mentioned earlier, although the first autochthonous cases were reported in 1909, leishmaniasis only became a public health concern in the Americas three decades later (9).

Living in the biggest endemic leishmaniasis country in the New World, Brazilian indigenous populations may be particularly exposed to leishmaniasis and other vector-borne diseases due to a combination of demographic and geographic factors. According to the 2010 census, Brazil has approximately 8,20,000 indigenous persons (61.47% in rural and natural areas) living in 505 reservations that cover 1.064 million square kilometers or 12.5% of the country (14). Despite its relatively small size, Brazil's indigenous population has enormous ethnic and linguistic diversity, with more than 300 indigenous ethnic groups and over 200 native languages, some of the highest indigenous diversity worldwide (15, 16).

Accordingly, the Brazilian government enacted policies in the 2000s and 2010s establishing the use of traditional territories (17). As a result, about 13% of the Brazilian territory is currently demarcated as federal indigenous lands (18). In addition, the geographic distribution of indigenous populations and reservations overlaps areas with endemic visceral leishmaniasis in Brazil. Although leishmaniasis has been described in indigenous populations of Brazil, the sample sizes and diagnostic tests applied in studies vary widely; moreover, studies have only been conducted in three main states: Mato Grosso, Minas Gerais, and Roraima.

As the largest territorial state in central-western Brazil, Mato Grosso underwent an entomological survey conducted from 2006 to 2008 and involving 25 indigenous villages, with a *Lutzomyia whitmani* identified in a total of 1,271 of 4,424 individuals surveyed (28.7%) and *Lutzomyia longipalpis* identified in 1,044 (23.6%) (19). An abundance of sand flies was recorded in the area, associated with the Cerrado/Amazon transition area and an expansion of mining, logging, and agriculture activities (19). Another study in Mato Grosso surveyed 25 other indigenous villages over an area of 1,68,000 hectares using a PCR testing approach, finding a positivity rate of 3/372 (0.8%) for *L. infantum* (20). Although the latter study lacked sandfly trapping, *Lu. longipalpis* and *Lu. cruzi* have been recorded in Mato Grosso. Human cases of VL in the state were linked to natural and peridomiciliary areas, with dog and livestock populations acting as reservoirs and blood-meal sources, respectively. Finally, the authors linked increased sandfly activity to an uptick in agricultural and mining activities (20). A more recent study also conducted in urban areas of the central-western Brazilian region revealed an abundance of *Lu. longipalpis* throughout 2017–2019, suggesting plasticity under a tropical regime of temperature, humidity, and wind speed (21). Another study has assessed the leishmaniasis status of humans and dogs in indigenous communities located in the states of Mato Grosso and Tocantins (22, 23). An IFAT analysis found a seropositivity rate of 2/470 (0.4%) in humans, while an ELISA test for *Leishmania infantum* in dogs resulted in a seropositivity rate of 28/327 (8.6%). The latter analysis also found that 20 (6.1%) dogs were seropositive for *Leishmania amazonensis* (23). Despite relatively low prevalence at the time, the study concluded that the serological status should be further investigated due to differences in serological methods. Finally, a case report of a 2-year-old indigenous child with Down's syndrome described a clinical CL patient with ulcerated facial lesions who was attended to at a local hospital in Tocantins (22).

The biggest southeastern Brazilian state, Minas Gerais, has shown that 333/359 (92.8%) of the cases of American tegumentary leishmaniasis (ATL) reported in the region over a period of 11 years were reported in the 33 villages of the Xakriabá Indigenous Reserve (24). The presence of vectors was also observed, with the most abundant species in the rural and periurban study area being *Lu. Longipalpis* and *Ny. Intermedia*, demonstrating sandflies' adaptation to different types of anthropic environments (25). *Lutzomyia longipalpis* and *Ny. intermedia* are both anthropophilic species and recognized vectors of *L. infantum* and *L. braziliensis*, respectively. These two sandfly species were also the most abundant species in peridomicile areas, though not in natural areas/trails surveyed in the Xakriabá Indigenous Reserve (24). An ecological study has suggested that the high ATL prevalence in this region may have been triggered by a large number of wild reservoirs and high deforestation rates (26). Thus, the peridomicile areas in these studies exhibit certain key aspects that exacerbate leishmaniasis exposure: domestic animals as food sources for female sandflies, fruit trees and vegetables as shelter, and organic material for immatures (24). Another study in the Minas Gerais revealed a 46% overall infection rate in dogs in the Krenak indigenous community (27). As dogs were mostly asymptomatic (27.3%) and oligosymptomatic (54.5%), such findings may reflect the ineffectiveness of dog culling based on low-sensitivity diagnostic tests as undetected animals remain in the area and maintain the *Leishmania* cycle (27).

The most septentrional and deep-forest state of Roraima has shown the highest percentage of the indigenous population (11.0%) when compared to 26 other Brazilian states, accounting for approximately 50,000 people (14). In Roraima, from 1989 to 1993, a total of 82 indigenous people and 390 dogs tested seropositive for *Leishmania* spp. in a survey of 74 different locations, probably related to massive immigration from endemic areas of northeastern Brazil to mining areas near indigenous reservations since the 1980s (28). The presence of *Lu. longipalpis* and leishmaniasis in Roraima is recent, with the first report in 1989 (28). Despite a lack of updated information, an international newspaper report in 2021 mentioned that the Yanomami people constantly suffer from the advance of illegal mining across contiguous forest areas between Amazon state and Venezuela (29). Yanomami are the most numerous indigenous group in Roraima, with approximately 28,100 individuals, and accounted for 11/27 (40.7%) of infected persons in an overall 313 indigenous CL cases between 2013 and 2017 (30). Male indigenous people were considered more likely to test positive due to higher phlebotomine exposure during activities in forest areas, including gathering, cropping, fishing, and hunting (30).

The Brazilian ecological pattern of leishmaniasis among the indigenous populations, along with environmental characteristics and exploratory activities, have also been associated with the migratory behavior of some ethnic groups, visiting relatives in other villages, or being forced to migrate due to land reallocation by the Brazilian government (31). Such movements of indigenous peoples with families and dogs may increase the risk of *Leishmania* spp. exposure and spread (28). Neither wild nor domestic dogs were part of pre-Columbian indigenous cultures in Brazil. However, dogs have taken on significant importance for indigenous communities, including for hunting and protection, along with other domestic, exotic animals such as chickens (32). Although *Lu. Longipalpis*' preference for biting dogs and chickens instead of humans has been shown in Brazil and Colombia; its presence in such peridomiciliary areas may increase human exposure to the disease. Further studies should be conducted to fully establish the role of introduced domestic animals in the *Leishmania* cycle in indigenous natural areas (28).

Although the second biggest northern Brazilian state, a recent study conducted in Pará was not included in this review due to a lack of original data (used information from the Brazilian Ministry of Health), findings included a single confirmed CL case among 183 surveyed indigenous people (0.5%), with a direct relationship between the increased CL cases and deforestation in the study area (33). Although the study did not directly contribute to the leishmaniasis survey in the present review, the authors established the “presence of cases in protected areas and a great epidemiological silence in indigenous lands,” indicating the absence of surveys in indigenous areas, even at present (33). Moreover, the authors hypothesized that such low case frequency of indigenous leishmaniasis might have been underestimated due to unsatisfactory attention by the local indigenous healthcare associated with access difficulties such as distance and infrastructure conditions. The study concluded that this lack of epidemiological information should be further investigated as an opportunity for combating the social, environmental, and public health liabilities historically established in the indigenous lands of the Amazon region. Finally, the study concluded that “this epidemiological silence reflects the fragility of these human populations and entails tragic outcomes related to their health conditions” (33).

As the fourth biggest country in Latin America (after Brazil, Argentina, and Peru), Colombia reported a VL prevalence study on *Leishmania chagasi* infection and risk factors in an indigenous community in the municipality of Coyaima (34). Of 385 indigenous people surveyed, 73 (19.0%) tested positive on the *Leishmania* skin test, 21/454 (4.6%) were positive on the *Leishmania* IFAT, and 23/454 (5.1%) tested positive on the *Leishmania* ELISA (34). The observed *Leishmania* seropositivity in indigenous people increased with age, and was higher among those affected by housing risk factors, though no effect of gender on leishmaniasis positivity was observed (35). The study concluded that continuous monitoring would be necessary to evaluate the long-term impact of housing risk factors on human leishmaniasis (34).

In another VL survey of Colombia, five rural areas of the Coyaima municipality reported no indigenous children under 5 years of age seropositive for *Leishmania infantum* antigens using the IFAT test. However, the positivity was 68.5% (185/270) among dogs (35). Despite 130/270 (48.1%) households having insecticide-impregnated bed nets, less than half of 112/270 (41.5%) referred to the knowledge of disease and traditional medical services, contributing to infection due to mal-information (35). The study concluded that control measures must be implemented to interrupt reservoir–vector–human transmission, including improving protective habits, living conditions, and the environment (35). In such a scenario, indigenous communities have been mostly built in poor traditional households with no mosquito-net protection, mostly located in environmentally protected areas, favoring vectors and reservoirs.

In western Colombia, a more recent study examined CL in the Pueblo Rico municipality, finding that 503/539 (93.3%) leishmaniasis-infected persons lived in dispersed rural areas and that 274/539 (50.8%) were Emberá (aboriginal Americans) indigenous persons (36). At the time, Pueblo Rico had a high rate of acute and chronic malnutrition in children under the 5-year population, along with the highest infant mortality rate due to malnutrition in the region (2.3 Cases/1,00,000 inhabitants/year) (36). In summary, the study concluded that leishmaniasis has mainly affected indigenous and rural populations with limited access to health services (36).

In Guyana, another Amazonian country, a recent study on various diseases in a southern indigenous Waiwai reserve showed that only one of 20 dogs surveyed (5.0%) had antibodies against *L. infantum*. The dog in question had been brought as an adult from northwestern Brazil and may have been infected elsewhere (37). Since dogs have been recognized as a major reservoir for *L. infantum* infections, the study shows the importance of dog movement among indigenous communities, particularly along international borders (37).

Effective diagnosis and treatment of VL and CL have historically persisted as a challenge in the New World, reflecting that disease remained neglected and that certain strategies need to be applied locally in a holistic approach to control the disease transmission. An association of VL and CL occurrence in remote areas, lack of a highly sensitive rapid test for prompt and reliable diagnosis, and a complex treatment regimen with variable sensibility may have altogether impaired the proper disease management worldwide (38). Not surprisingly, a recent study has shown resistance to meglumine antimoniate by *L. braziliensis* in South American countries, as previously observed in India for *Leishmania donovani* and *L*. (*Viannia*) subgenus (39). Natural resistance has also been reported in clinical and *in vitro* use of miltefosine for VL by *L. infantum* in Brazil, despite being considered appropriate for ATL treatment (40, 41). *Leishmania* sp. resistance to first- and second-line drugs has fluctuated in several regions of Colombia over 30 years, with miltefosine and meglumine antimoniate presenting drug resistance varying from 0 to 69% for *L. guyanensis, L. braziliensis*, and *L. panamensis* (42). Finally, according to a recent systematic review, CL treatment in the New World has shown a high frequency of side effects in first and second-line drugs with no defined criteria for collateral severity (41).

## Leishmaniasis in indigenous people of Old World

Considered a poverty-related disease in the Old World, VL has registered the highest incidences in southeast Asia and sub-Saharan Africa (43, 44). Despite the worldwide survey and current VL distribution confined to certain geographical localities of the present study, vector spreading in new areas has been observed. It may impact future investigations (45). In such regions, particularly in Eastern Africa and Indian subcontinent countries (ISC), *Leishmania donovani* has been transmitted human-to-human by *Phlebotomus* spp. sandflies, contrasting with the animal–human transmission of *L. infantum* in the New World (46). Widespread CL occurrence has also been reported in tropical and subtropical areas of ISC, the Middle East, Mediterranean seashore, Arabian Peninsula, Africa, and Asia (47, 48). In these areas, CL has been caused by *Leishmania tropica* as anthroponosis, *Leishmania major* as zoonosis, *Leishmania aethiopica*, and *L. infantum* with restricted distribution and transmission by *Phlebotomus* spp. sandflies (47). In addition, new CL cases in ISC have been caused by an atypical phenotype of *Leishmania donovani* (49), with dermotropic tropism and correspondent genetic variations yet to be fully clarified (49, 50). Although more than 50% of VL global cases for the last decade were reported in ISC, such as India, Bangladesh, and Nepal, VL incidence and mortality rates have recently decreased in India (51). Such reduction has been attributed to governmental insecticide spraying in endemic areas, the use of rK39 rapid diagnostic tests, and combined therapy protocols such as miltefosine and liposomal amphotericin B (52, 53). Finally, a previous study has shown that miltefosine combined with paromomycin was a cost-effective therapeutic approach in endemic areas of India, which should be reproduced in other VL endemic areas such as East Africa and Brazil (54).

In addition to sand flies, biting midges have been reported to be the main vectors of these emerging pathogens, including the new recently reported subgenus Mundinia, which comprises human-infective (*Leishmania orientalis* and *Leishmania martiniquensis*) and non-human infective species (*Leishmania enriettii* and *Leishmania macropodum*). The widely distributed biting midge species *Culicoides sonorensis* was able to transmit *L. orientalis, L. martiniquensis*, and *L*. sp. Ghana under experimental conditions to biting location in BALB/c mouse (55). Both *L. orientalis*, and *L. martiniquensis* have been identified in autochthonous VL and CL cases, mostly in immunocompromised individuals of Southeast Asian countries, including Thailand and Myanmar (56–58). As these two countries lack representative leishmaniasis survey studies by rapid immunodiagnostic tests and PCR in the general population, prevalence and incidence remain to be established (57), which may also impair diagnosis in indigenous populations of remote areas. Essentially, these two species had been previously described as “*L. siamensis”* for years because they were diagnosed in indigenous Thai and Myanmar patients who had no history of abroad traveling (59, 60). The remaining cases resulted in *L. martiniquensis* and *L. orientalis*, and the invalid nomenclature of the “*L. siamensis”* species term was discontinued (56, 61). In addition, other local Mundinia species (*L*. sp. from Ghana and *L*. sp. from Namibia) have also been reported in West and South African countries (62, 63). As members of this subgenus have been distributed worldwide, speculation was made that genetic differences from their common ancestor had occurred before the Gondwana separation (6). Once more, the present study has focused on indigenous rather than native populations as the last comprises all born citizens infected as autochthonous cases.

In Iran, leishmaniasis has been an important public health concern and ranked as the second most prevalent vector-borne, only surpassed by Malaria (64). An early study focused on the clinical outset of 1,250 patients attended within 2 years, observing no associated risk factor with age, sex, and clinical lesion features (65). However, nonindigenous patients presented more lesions, which were more acute and severe when compared to those of indigenous patients (65). Authors had no explanation at the time for why nonindigenous patients presented more severe lesions and in greater numbers but hypothesized that nonindigenous people were digging workers exposed outdoors without protection to infected vectors in more infected areas (65). A second explanation was that indigenous people may have developed mild immunity during a long period of exposure time (65).

In Taiwan, a single leishmaniasis study of three aboriginal cases has been described and alerted for the persistence of the disease in the country's low-altitude mountains, despite the sporadic status at the time (66). In addition, such a rare occurrence has led to misdiagnosis, particularly when patients have not traveled internationally to endemic areas (66).

As previously mentioned, emerging resistance to first-line drugs to VL and CL has been reported in South America, Europe, and Asia, associated with a lack of antileishmanial drugs clinically approved, toxicity effects in systemic treatment, temperature-controlled transport and storage, high costs, and absence of clinically approved antileishmanial vaccines (67). Although a study in Iran has shown *in vitro* sensibility to miltefosine and paromomycin in patients with CL, *L. tropica* showed resistance to pentavalent antimony, highlighting the difficulties in drug selection effectiveness (68).

## Final considerations

This is the first study focused on leishmaniasis in indigenous populations. Although such communities may be similarly exposed to leishmaniasis (and other vector-borne diseases) as their surrounding rural or remote populations within endemic areas, indigenous communities may differ in discrimination, ethnicity and social vulnerability, cultural and behavioral practices, and health assessment due to legal regulations among countries. Most new leishmaniasis cases worldwide have been reported in countries with indigenous communities located within endemic areas. Such indigenous communities have been mostly built in poor traditional households with no mosquito-net protection, mostly located in environmentally protected areas, favoring vectors and reservoirs. The presence of leishmaniasis cases surrounding such indigenous areas indicated a high risk of infection, which may have been historically underestimated due to a lack of surveillance, even at present. The lack of surveys in indigenous populations in endemic areas worldwide may reflect the difficulties faced by the local health services, in addition to other obstacles such as cell phone or internet coverage, offroad access, climate, distance, and other logistical conditions.

As a limitation, the keyword “indigenous” may have picked up inappropriate articles for this review, especially the Old World cases. For example, “indigenous” may be used as “autochthonous” in the article on Taiwanese cases. Similarly, the terms “indigenous and nonindigenous people” may not be clear in the article on Iranian cases. On the other hand, the definition of “indigenous people” has been very clear in the New World cases. Thus, the differences between the New World and Old World cases may have made leishmaniasis a unique perspective in indigenous people in the New World. However, the inclusion of Old World studies may serve as a warning for future discrimination and standardization of terms in the Old World, as such vulnerable originating populations in this region may be misidentified and, therefore, underdiagnosed. Finally, the study herein was limited to keywords in English only, which may have impaired the survey in the native languages of endemic countries.

Moreover, both human and animal leishmaniasis in indigenous populations around the world may reflect that this infectious disease has remained neglected and that certain strategies in a holistic approach need to be applied locally to control the disease transmission. Regardless, indigenous populations worldwide should always be considered as a human heritage and respected as vulnerable and traditional communities which deserve the government's protection and a minimum of basic sanitary and educational infrastructure conditions.

## Author contributions

LK, AB, and FF: conceptualization. LK, LB, AB, and FF: original draft preparation, writing, reviewing, and editing. FF: supervision. All authors contributed to the article and approved the submitted version.

## Supplementary material

Supplementary Figure 1 Global indigenous lands and poverty indicators.

Supplementary Figure 2 Climate and average precipitation global data illustrate the regions where leishmaniasis is endemic.

Supplementary Material 1 Selected databases for construction of Figure 1, Supplementary Figures 1, 2.
