# Supplementary figures and images for: Leishmania spp. in indigenous populations: A mini-review
Source: Front Public Health. 2022 Dec 22;10:1033803. doi: 10.3389/fpubh.2022.1033803 (PMC9815601; doi:10.3389/fpubh.2022.1033803)

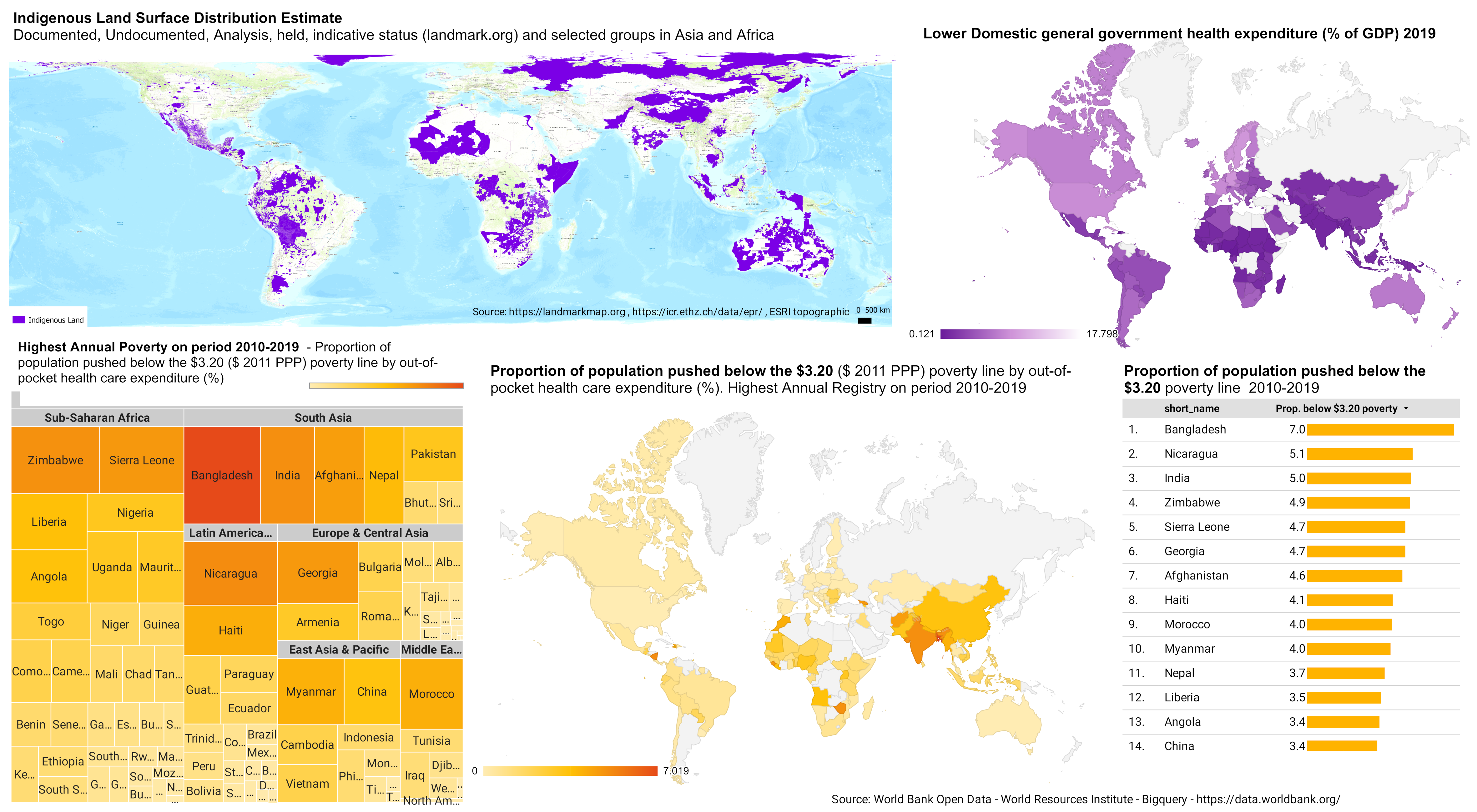

Supplement: Supplementary Figure 1 — Global indigenous lands and poverty indicators. [file Image_1.TIF]

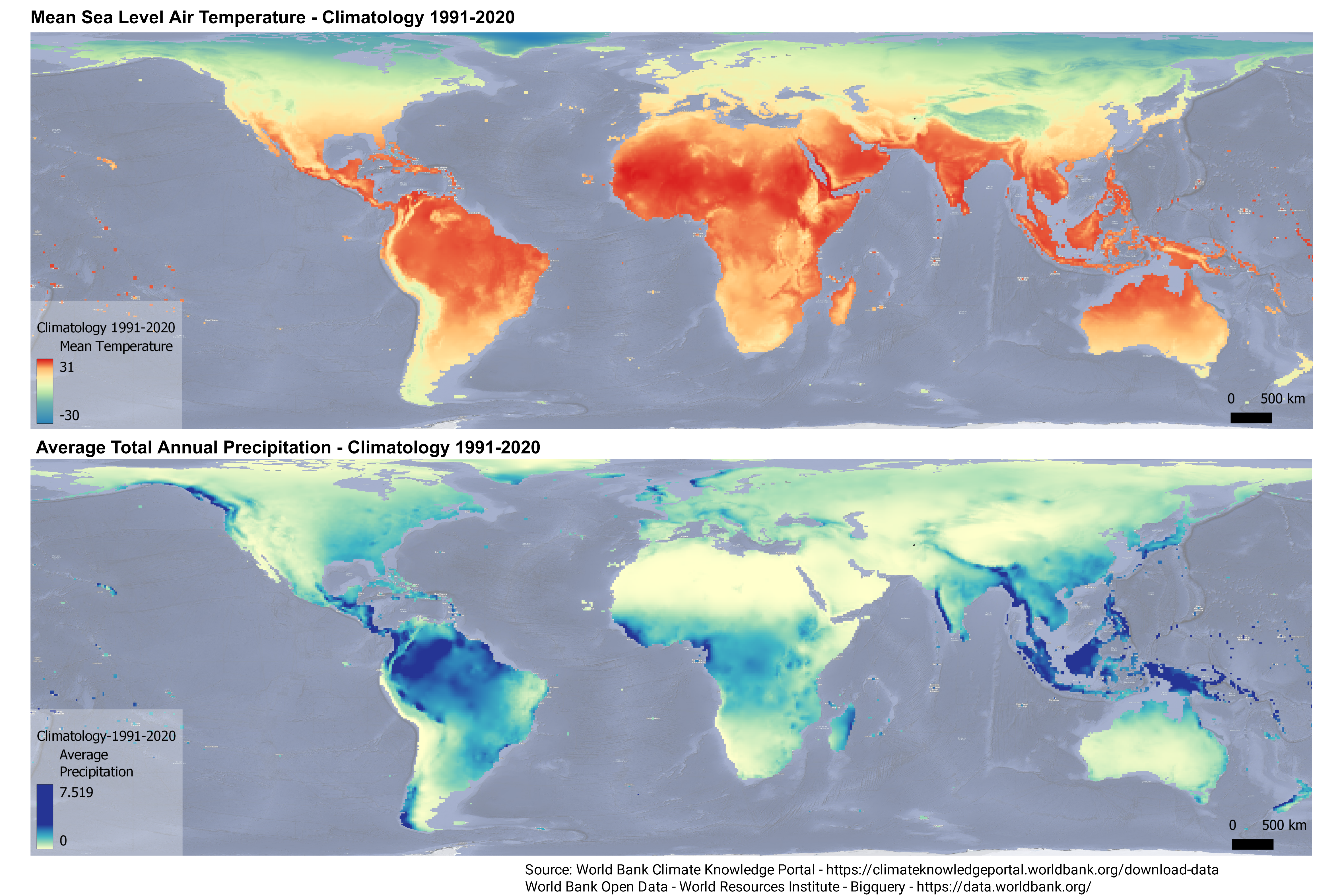

Supplement: Supplementary Figure 2 — Climate and average precipitation global data illustrate the regions where leishmaniasis is endemic. [file Image_2.TIF]
