# Supplementary material for: Leishmania spp. in indigenous populations: A mini-review
Source: Front Public Health. 2022 Dec 22;10:1033803. doi: 10.3389/fpubh.2022.1033803 (PMC9815601; doi:10.3389/fpubh.2022.1033803)
Supplement: Supplementary Material 1 — Selected databases for construction of Figure 1, Supplementary Figures 1, 2. [file Data_Sheet_2.DOCX]

Supplementary Material

# Supplementary Table 01. *Leishmania* spp. in vectors of indigenous populations worldwide.

| Sandflies species | *Leishmania* spp. detection | Comment(s) (including related disease[s]) | Geographical distribution |
| --- | --- | --- | --- |
| *Brumptomyia brumpti; Lutzomyia andersoni; Lutzomyia antunesi; Lutzomyia aragaoi; Lutzomyia barrettoi barrettoi; Lutzomyia begonae; Lutzomyia brasiliensis; Lutzomyia carmelinoi; Lutzomyia carrerai carrerai; Lutzomyia chagasi Lutzomyia choti; Lutzomyia christenseni; Lutzomyia claustrei; Lutzomyia cruciata; Lutzomyia cruzi; Lutzomyia davisi; Lutzomyia evandroi; Lutzomyia flaviscutellata Lutzomyia furcat; Lutzomyia goiana; Lutzomyia hermanlenti; Lutzomyia inflata; Lutzomyia lenti; Lutzomyia longipalpis; Lutzomyia longipennis; Lutzomyia longispina Lutzomyia lutziana; Lutzomyia octavioi; Lutzomyia pinottii; Lutzomyia runoides; Lutzomyia sallesi; Lutzomyia saulensis; Lutzomyia shannoni; Lutzomyia sordellii Lutzomyia teratodes; Lutzomyia termitophila; Lutzomyia walkeri; Lutzomyia whitmani* |  | *L. whitmani* and *L. flaviscutellata* are a recognized vector of ATL, and *L. longipalpis* and *Lutzomyia cruzi* of VL in Brazil. | 2006-2008/ America- Brazil (18) |
| *Lu. longipalpis*; *Ny. intermedia*; *Martinsmyia minasensis*; *Lutzomyia cavernicola* | - | - | 2008-2009/ America- Brazil (23) |
| *Evandromyia apurinan*; *Nyssomyia umbratilis*; *Nyssomyia yuilli yuilli*; *Psychodidae davisi*; *Sciopemyia servulolimai* | *Leishmania* (*Leishmania*) *amazonensis, Leishmania* (*Viannia*) *braziliensis* | - | 2012/ America- Brazil (40) |
| *Ny. intermedia; Lu. longipalpis; Migonemyia migonei and Pintomyia pessoai* | - | - | 2015-2016/ America-Brazil (41) |
