# Supplementary material for: Leishmania spp. in indigenous populations: A mini-review
Source: Front Public Health. 2022 Dec 22;10:1033803. doi: 10.3389/fpubh.2022.1033803 (PMC9815601; doi:10.3389/fpubh.2022.1033803)
Supplement: Supplementary file 5 [file Data_Sheet_3.PDF]

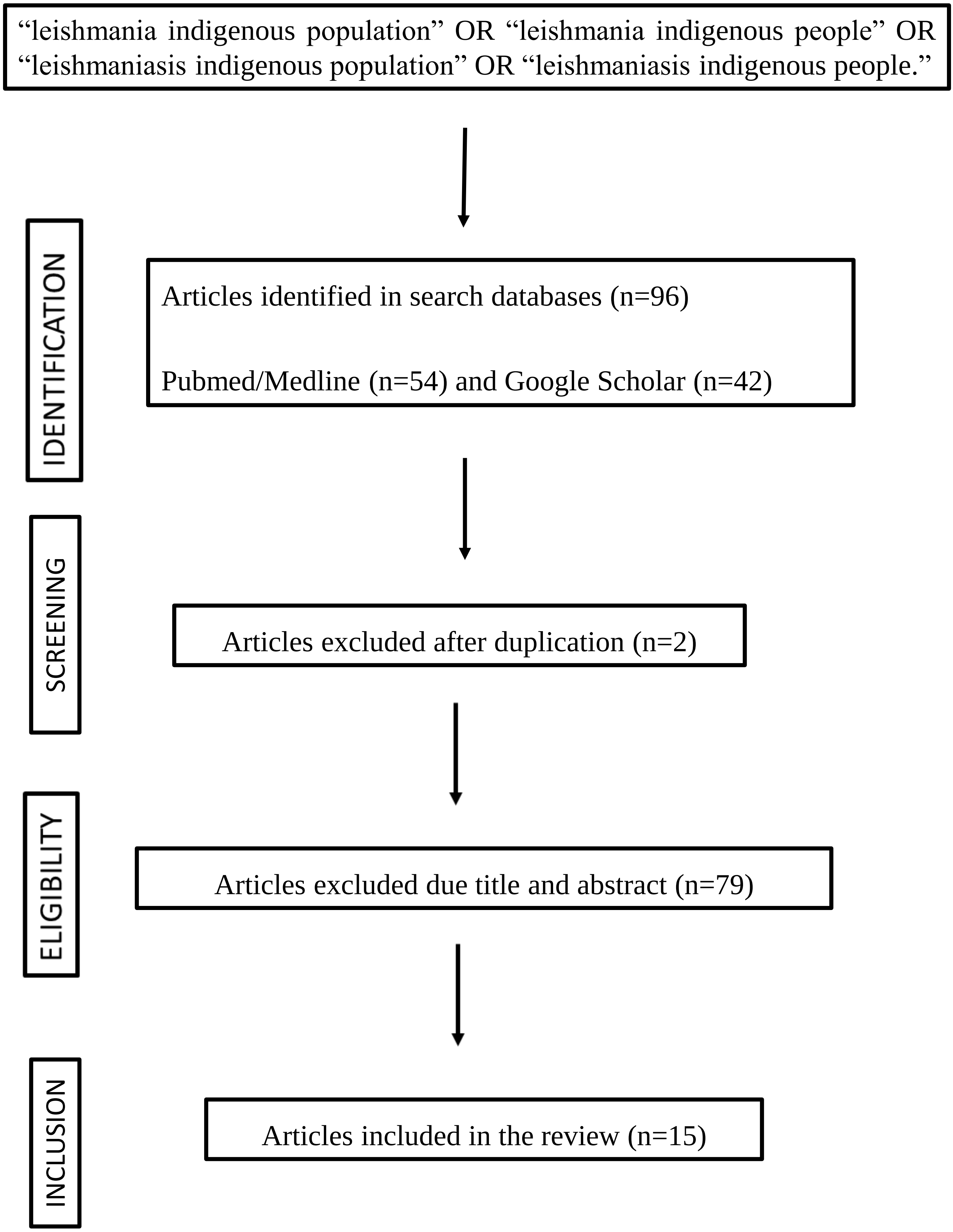
"leishmania indigenous population" OR "leishmania indigenous people" OR "leishmaniasis indigenous population" OR "leishmaniasis indigenous people."
IDENTIFICATION
Articles identified in search databases (n=96)
Pubmed/Medline (n=54) and Google Scholar (n=42)
SCREENING
Articles excluded after duplication (n=2)
ELIGIBILITY
Articles excluded due title and abstract (n=79)
INCLUSION
Articles included in the review (n=15)
